# Association of water, sanitation, hygiene and food practices with enteric fever in a paediatric cohort in North India

**DOI:** 10.1136/bmjpo-2021-001352

**Published:** 2022-04-26

**Authors:** Nonita Dudeja, Bireshwar Sinha, Nidhi Goyal, Alok Arya, Anitha Revi, Ankita Dutta, Deepak More, Aparna Chakravarty, Chandra Mohan Kumar, Temsunaro Rongsen-Chandola

**Affiliations:** 1 Division of Infectious Diseases, Centre for Health Research and Development, Society for Applied Studies, New Delhi, India; 2 Clinical and Research Laboratories, Society for Applied Studies, New Delhi, India; 3 Department of Paediatrics, Hakeem Abdul Hameed Centenary Hospital, Hamdard Institute of Medical Sciences and Research, New Delhi, Delhi, India

**Keywords:** epidemiology

## Abstract

**Background:**

Our aim was to assess the association of water, sanitation and hygiene (WASH) and food practices with culture-confirmed enteric fever in children <15 years of age.

**Methods:**

We followed a cohort of 6000 children from an urban low socioeconomic neighbourhood in South Delhi for 2 years to estimate burden of culture-confirmed enteric fever. Risk ratios (RRs) were estimated to study the association between WASH practices and enteric fever. We assessed the microbiological quality of drinking water and conducted geospatial analysis to evaluate the distribution of enteric fever cases around households with contaminated drinking water.

**Results:**

A total of 5916 children in 3123 households completed survey. Piped water (82%) was the major source of household drinking water. One-third (32%) of the households treated water before consumption. Almost all households had sanitary toilets (99.9%) and 16% used shared toilets. Consumption of food from street vendors and unnamed ice creams more than once a week was observed in children from 12.7% and 38.4% households, respectively. Eighty culture-confirmed enteric fever cases were reported. The risk of enteric fever was 71% higher in children belonging to households having food from outside once a week or more (RR 1.71, 95% CI 1.00 to 2.94). The RR for enteric fever in children living in households with availability of safe drinking water was 0.75 (95% CI 0.45 to 1.26). We found that 14.8% of the households had presence of coliforms or *Escherichia coli* in their household drinking water. The odds of having a case of enteric fever within a 5 and 25 m buffer zone around households with contaminated drinking water were 4.07 (95% CI 0.81 to 20.5) and 1.44 (95% CI 0.69 to 3.00), respectively.

**Conclusion:**

In addition to WASH practices, optimal food hygiene may have a role in urban low socioeconomic population to control enteric fever.

**Trial registration number:**

CTRI*/*2017/09/009719.

**What is known about the subject?**

- Enteric fever is common in children and endemic in low-income and middle-income countries and may be associated with water, sanitation and hygiene (WASH) factors and food practices.
- Recently, there have been global and country-specific efforts to improve access to safe water and sanitation facilities.

**What this study adds?**

- In the current context, we examined the strength of association of enteric fever with WASH and food practices in an urban slum in North India.
- Most households had improved source of drinking water and sanitary toilet. Frequent consumption of food from street vendors had a substantially higher risk of culture-confirmed enteric fever in children.
- Beyond good WASH practices, education on optimal food hygiene practices in low-income communities might have an additional role to control enteric fever.

## Introduction

 Low-income and middle-income countries have high burden of enteric fever, with South Asia and sub-Saharan African countries accounting for majority of the cases.^1^ Estimates from a meta-analysis showed that the population-based pooled incidence of typhoid and paratyphoid in India was 377 (178–801) and 105 (74–148) per 100 000 person years, respectively, with highest incidence among children aged 2–4 years.^2^ In an earlier cohort in Delhi, the incidence rate of typhoid per 1000 person-years was 27.3 among under-5 children, 11.7 in 5–19 year olds, and 1.1 among participants aged 19 and 40 years.^3^

Risk of enteric fever seems to be higher in populations with poor accessibility to safe water and sanitation facilities.^4^ A recent systematic review on the association between water, sanitation and hygiene (WASH) factors and typhoid fever revealed that improved water source (OR 0.73, 95% CI 0.56 to 0.95), treated water (OR 0.59, 95% CI 0.45 to 0.75), availability of household latrine (OR 0.87, 95% CI 0.68 to 1.11) and good hygiene (OR 0.52, 95% CI 0.40 to 0.67) were associated with lower risk of typhoid fever, while open defecation (OR 0.99, 95% CI 0.84 to 1.18) and unsafe waste management (OR 1.56, 95% CI 1.25 to 1.95) were associated with higher risk of typhoid.^5^

In India, less than 50% of the population has access to safely managed drinking water.^6^ However, there is an improvement in the availability of basic sanitation (from 38% in 2014 to 95% in 2019) following implementation of Swachh Bharat Mission (2014).^7^ The studies on the association of WASH and enteric fever from Indian settings included in the review by Brocket *et al* were published in the early 2000s.^8^,^10^ Also, there was a lot of heterogeneity in the burden of enteric fever across different states ranging from 215 per 100 000 PY in Kolkata to 980 per 100 000 PY in New Delhi.^11^,^14^ Multiple initiatives during these years have affected the WASH and food hygiene practices as well as immunisation schedules across different states in the country. Contrary to the speculations of a lower burden, our recent study reported a high burden of enteric fever, that is, 703.7 (95% CI 560.5 to 874.7) per 100 000 PY.^15^

In the present scenario, with improved access to water and sanitation, the role of WASH practices towards disease burden of enteric fever remains to be reasserted. Using the data from our paediatric longitudinal cohort in Delhi,^16^ we aimed to estimate the association between WASH and food practices with culture-confirmed enteric fever. As an exploratory exercise, we also aimed to study the spatial distribution of enteric fever cases and contaminated drinking water in the community by using geographical information system (GIS).

## Methods

### Study design

This study was part of the ‘Surveillance of Enteric Fever in India (SEFI)’ paediatric cohort, conducted for estimation of enteric fever burden in children across four sites in India. The protocol of the SEFI study has been published previously.^16^ The North Indian site comprised adjoining blocks of Sangam Vihar, a low-income urban neighbourhood in the south district of New Delhi.

### Data collection

We conducted a door-to-door household survey to identify eligible children aged 6 months–14 completed years. Six thousand eligible children who consented to participation were enrolled and followed up weekly for 24 months from enrolment or until the child attained the age of 15 years (whichever was earlier). We conducted geotagging of all enrolled participants and households. In the event of fever, our team made daily contacts until the end of the fever episode. Blood culture was performed if fever lasted for three or more consecutive days. WASH and food hygiene practices were assessed using a structured interview schedule that was administered during home visit in the study households. The WASH questionnaire was based on a modified version of the WHO document on core questions on drinking water and sanitation for household surveys.^17^ Information related to food practices was obtained for the eldest enrolled child in the household.

To estimate the prevalence of microbiologically safe drinking water^18^ consumption among households in the study area, we conducted a subsurvey in 108 households selected by simple random sampling in December 2019. The sample size for this exercise was calculated assuming that 50% of households in the community would have access to microbially safe drinking water with a relative allowable error of 20%.^19^ We collected drinking water samples (1.5 L) from the drinking water storage point in these households to test for the most probable number of coliform per 100 mL, pH and total dissolved solids. The laboratory tests were conducted in TÜVs (Technischer Überwachungsverein) (TUV SUD), Gurgaon, Haryana, India. Presence of coliforms in drinking water was labelled as *contaminated drinking water*.

### Data management

Data were collected on tablets with built-in GIS using Android application package ‘EntericFev’, developed in-house by the SEFI team and stored in a secured Amazon cloud-based server. Data were monitored using a dashboard-based system; weekly reports were generated and reviewed. Quality checks were conducted by a central independent team of experts.

### Data variables and analysis

**Figure 1:**
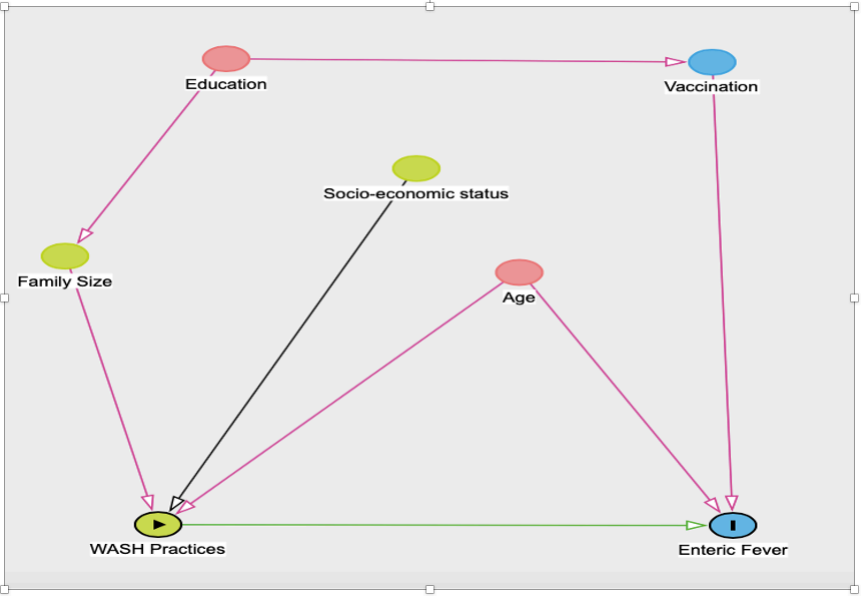
Directed acyclic graph model illustrating factors related to WASH (exposure) and enteric fever (outcome). WASH, water, sanitation and hygiene.

**Figure 2:**
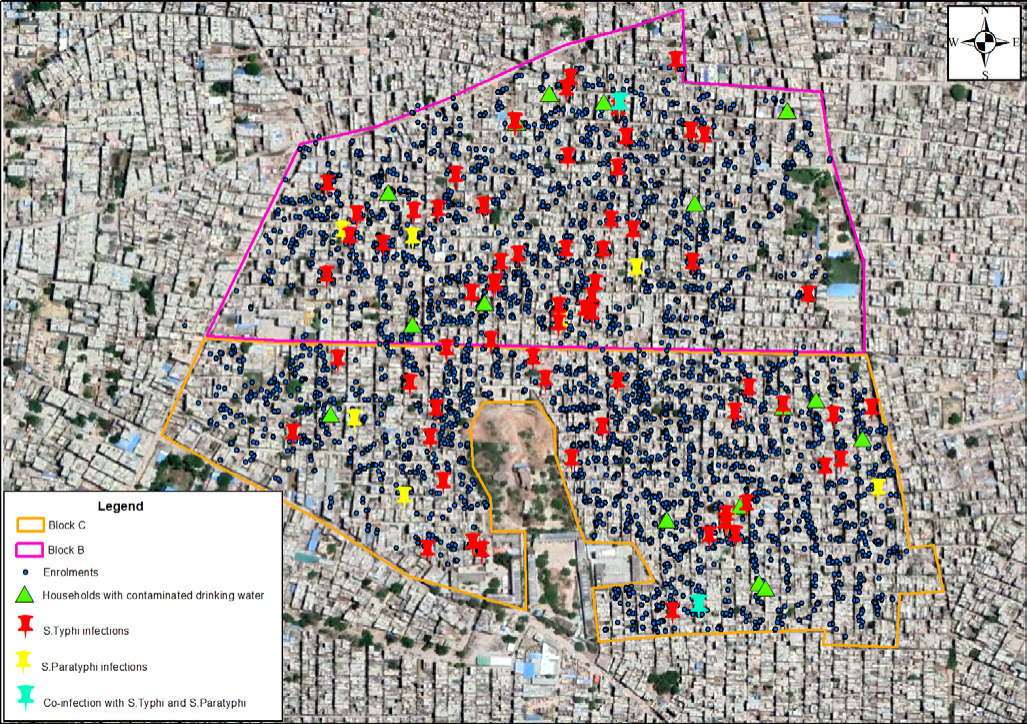
Geographical information system map showing cases of enteric fever in the study area and the households with contaminated drinking water.

Data were analysed by Stata V.16 software. Mean and median were calculated for continuous variables, while proportions were calculated for categorical variables. We conducted a child-based analysis to study the association between WASH practices and enteric fever. To account for the design effects of household clustering, when more than one child were included in the cohort from a household, we used Stata’s robust variance estimator (cluster) option. *Improved source of drinking water* was defined as use of piped water system or bottled water. *Adequate water treatment* was defined as treatment of water by boiling, bleach/chlorine or filtration. *Safe drinking water* in a household was defined as presence of improved source of drinking water along with practice of adequate water treatment. An *improved toilet facility* included flush toilets and pit latrines. *Safe sanitation* was defined as improved toilet facility with no sharing of toilets. Consumption of cooked foods, along with rare consumption of outside food (consumption of food from street vendors and/or unnamed ice creams less than once a week), was labelled as *good food practices*. Univariable analysis was done to see the association between WASH and food practices with incidence of enteric fever. Based on the directed acyclic graph (DAG) conceptual model (daggity.net), age, education, family size and receiving typhoid vaccine were identified as the potential confounders. We included the covariates in our final multivariable model which were either suggested by the conceptual DAG model or significant at p<0.25 in univariable analysis (figure 1). The cases of typhoid and paratyphoid along with the households with contaminated drinking water were plotted using ArcGIS V.10.8 on a Google Earth base map of July 2021 for the study area^20^ (figure 2). We estimated Moran’s index to assess clustering of enteric fever cases in the area.^21^ We conducted a buffer analysis to estimate the odds of a case of enteric fever within a coverage area of 5, 10 and 25 m radius around the study households with and without contaminated drinking water.^22^

### Patient and public involvement statement

Patients or the public were not involved in the design, conduct, reporting or dissemination plans of our research.

## Results

A total of 6000 children <15 years were enrolled; 31% were <5 years; 42% were between 5 years and 10 years; and 27% were between 10 years and 15 years of age. Around half of them were male (51%). Three-fourth (73%) of the children belonged to nuclear families, and 70.6% belonged to overcrowded households. The median monthly income was Rs 10 000 (IQR 8000–15 000). In this paediatric cohort, 13.7% had been vaccinated against typhoid, of which 95% received a polysaccharide vaccine, mostly from a government health facility (table 1).

**Table 1:** Baseline characteristics of the study population (N=5916)

Characteristic	n* (%)
Total number of households	3123
Female gender	2896/5916 (48.9)
Age group (years)	
<5	1850/5916 (31.3)
5–10	2469/5916 (41.7)
>10	1597/5916 (27.0)
Nuclear family	4301/5916 (72.7)
Family size: mean (SD) (N=5916)	5.7 (2.2)
Highest education in family as years of schooling	
Illiterate	66/5916 (1.1)
<5	114/5916 (1.9)
>5	5736/5916 (97)
Pucca house†	5862/5916 (99.1)
Proportion of children living in overcrowded households(>2.5 persons per living room)	4233/5916 (70.6)
Separate kitchen available	3770/5916 (63.7)
Monthly income in Indian national rupees: median (IQR) (n=5916)	10 000 (8000–15 000)
Received typhoid vaccine	820/6000 (13.7)
Type of typhoid vaccine received	
Polysaccharide	778/820 (94.9)
Conjugate/unclear	42/820 (5.1)
Vaccine received at a government facility	782/820 (95.4)

* Figures indicate n (%), unless indicated otherwise.

† Pucca house is a term that refers to housing in South Asia built of substantial material such as stone, brick, cement, concrete or timber.

The survey on demographic characteristics and WASH and food practices was completed in 3123 households with 5916 children. Piped water from the Delhi municipal corporation (82%) and bottled water (10%) were the major sources of household drinking water; around one-third (32%) of the households treated water before consumption. The most common method of purification was filtration (94%). Almost all the households had sanitary toilets, of which 21% had flush systems. Sharing of toilets with other families was observed in 16% of the households. Among children >3 years of age, 90% (4387/4903) were using toilets; 4% (194/4903) were disposing off stools in the garbage, while in 1.1% (53/4903), the stools were disposed-off in the drain/ditch. Consumption of uncooked food without washing was observed in 1.9% of the households. Consumption of food from street vendors and unnamed ice creams from street vendors more than once a week was noted in participants in 12.7% and 38.4% of households, respectively. (table 2)

**Table 2:** WASH and food practices (N=5916)

WASH parameter	n (%)N=5916
Drinking water-related practices	
Source of drinking water	
Piped water from Municipality	4845 (81.9)
Bottled water	605 (10.2)
Public tap/standpipe	226 (3.8)
Others*	240 (0.06)
Households treating water before consumption	1917 (32.4)
Methods of water treatment (N=1917)	
Filtration†	1809 (30.6)
Boiling	92 (1.5)
Others ‡	16 (0.3)
Improved source of drinking water	5158 (87.2)
Adequate water treatment	1907 (32.2)
Safe drinking water	1733 (29.3)
Sanitation practices	
Type of toilet facility used	
Pit latrines	4695 (79.3)
Flush type toilet	1220 (20.6)
Composting toilet	1 (0.1)
Children living in households with shared toilets	942 (15.9)
Child stool disposal practices	
Child used toilet/latrine	5046 (85.3)
Discarded into toilet/drain	505 (8.5)
Others§	363 (6.2)
Safe sanitation	4965 (83.9)
Food practices	
Practice of consumption of uncooked food without washing	111 (1.9)
Consuming ice cream (stick ice/unnamed ice creams from street vendors) once a week or more	2275 (38.4)
Eating foods from outside once a week or more	752 (12.7)
Eating uncooked food once a week or more	4117 (69.6)
Good food practices	1188 (20.0)

* Others included tanker trucks and tube well.

† 1166 (19.7%) used a candle filter, while 643 (10.9%) used an electric water purifier.

‡ Others included straining through cloth and storage.

§ Others included throwing into a garbage and left in the open.

WASH, water, sanitation and hygiene.

A total of 80 children had culture-confirmed enteric fever; 68 had *Salmonella typhi*; and 10 had *S. paratyphi*; and two children had coinfection. No enteric fever-associated deaths were observed. Spatial autocorrelation analysis suggested a clustering pattern for the typhoid fever cases in the study area (Moran’s Index 0.48, z-score 2.5, p=0.012); no clustering was suggestive for paratyphoid fever cases (Moran’s Index 0.16, z-score 0.28, p=0.778).

The risk of enteric fever was 1.71 times higher in children belonging to households eating food from outside once a week or more (adjusted risk ratio (RR) 1.71, 95% CI 1.00 to 2.94). The risk of enteric fever was 25% lower in children living in households with availability of safe drinking water (adjusted RR 0.75, 95% CI 0.45 to 1.26); however, the association was not statistically significant. The risk of enteric fever was 1.32 times higher in children living in households sharing toilets (adjusted RR 1.32, 95% CI 0.76 to 2.30; table 3).

**Table 3:** Unadjusted and adjusted relative risk for association between WASH parameters and enteric fever

WASH practices	No enteric feverN=5836, n (%)	Enteric feverN=80, n (%)	Unadjusted RR(95% CI)	P value	Adjusted* RR(95% CI)	P value
Water						
Improved source of drinking water†	5087 (87.2)	71 (88.8)	1.16 (0.58 to 2.31)	0.674	1.15 (0.58 to 2.30)	0.686
Adequate water treatment‡	1886 (32.2)	21 (26.3)	0.75 (0.46 to 1.23)	0.261	0.75 (0.45 to 1.23)	0.254
Safe drinking water§	1714 (29.4)	19 (23.8)	0.75 (0.45 to 1.25)	0.275	0.75 (0.45 to 1.26)	0.273
Sanitation						
Toilet sharing	926 (15.9)	16 (20.0)	1.32 (0.77 to 2.27)	0.317	1.32 (0.76 to 2.30)	0.322
Safe sanitation¶	4901 (84.0)	64 (80.0)	0.77 (0.44 to 1.32)	0.337	0.77 (0.44 to 1.33)	0.342
Food practices						
Consumption of ice creams once a week or more	2247 (37.9)	28 (35.0)	0.88 (0.56 to 1.39)	0.589	0.86 (0.55 to 1.36)	0.527
Eating food from outside once a week or more	736 (12.4)	16 (20.0)	1.74 (1.01 to 3.00)	0.044	1.71 (1.00 to 2.94)	0.053
Consumption of uncooked food once a week or more	4063 (68.6)	54 (67.5)	0.95 (0.60 to 1.51)	0.828	0.90 (0.56 to 1.43)	0.654
Good food practices**	1171 (20.1)	17 (21.3)	1.07 (0.63 to 1.83)	0.614	1.08 (0.64 to 1.85)	0.613

* Adjusted for age, education, family size and typhoid vaccine received.

† Improved source of drinking water was defined as use of piped water system or bottled water.

‡ Adequate water treatment was defined as treatment of water by boiling, bleach/chlorine or filtration.

§ Safe drinking water in a household was defined as presence of improved source of drinking water and practice of adequate water treatment.

¶ Safe sanitation was defined as improved toilet facility with no sharing of toilets. More than 99% of the study subjects belonged to households with an improved toilet facility.

** No consumption of uncooked food, no consumption of food from outside and no consumption of unnamed ice creams once a week or more.

RR, risk ratio; WASH, water, sanitation and hygiene.

We found that 16/108 (14.8%, 95% CI 8.7% to 22.9%) of the households had presence of coliforms in their household drinking water; all the 16 households had a coliform count of >100/100 mL of drinking water. The median total dissolved solids in drinking water was 130 mg/L (IQR 65–151) and the mean (SD) pH was 7.2 (0.5). Buffer analysis revealed that the odds of having a case of enteric fever in the surrounding radius of 5, 10 and 25 m in households with contaminated drinking water against households without contaminated drinking water were 4.07 (95% CI 0.81 to 20.5), 1.74 (95% CI 0.54 to 5.67) and 1.44 (95% CI 0.69 to 3.00), respectively (table 4).

**Table 4:** Odds of enteric fever within a buffer area of 5, 10 and 25 m radius around the study households with and without contaminated drinking water

Buffer zone (m)	Population	Total enrolled	Enteric fever positive	Enteric fever negative	OR(95% CI)
5	Children living in the buffer zone around HHs with contaminated drinking water	101	3	98	4.07 (0.81 to 20.5)
	Children living in the buffer zone around HHs without contaminated drinking water	402	3	399	
10	Children living in the buffer zone around HHs with contaminated drinking water	160	4	156	1.74 (0.54 to 5.67)
	Children living in the buffer zone around HHs without contaminated drinking water	695	10	685	
25	Children living in the buffer zone around HHs with contaminated drinking water	619	11	608	1.44 (0.69 to 3.00)
	Children living in the buffer zone around HHs without contaminated drinking water	1783	22	1761	

HH, household.

## Discussion

This study reports the association between WASH practices and incidence of enteric fever in children up to 15 years of age. Our findings suggest that consumption of safe drinking water and safe sanitation practices were associated with a lower risk of enteric fever, while sharing of toilets and eating food from outside were associated with a higher risk of enteric fever. However, the precision for some of these associations was wide and the association is non-significant. Nearly 15% of the households had presence of *E. coli* or coliforms in their household drinking water. Geospatial analysis revealed higher odds of a case of enteric fever in surrounding zones of households with contaminated drinking water as compared with those without contaminated drinking water.

In the present study, the possibility of selection bias was low, as all children from geographically contiguous areas in the study site were offered participation. Though the collection of data related to WASH was not recorded from all the households with eligible children, we could capture this information for 98.5% of the households. Water sampling and testing were done by trained manpower as per standardised guidelines. Active weekly contacts and incentivisation in the form of phone recharge were done to ensure reporting of fever episodes and to reduce loss to follow-up rates. Blood culture sensitivity is 60% to detect enteric fever, and therefore there is a possibility of some degree of underdetection of cases. We believe that unequal distribution of the undetected cases among the exposed and unexposed groups is unlikely, but the small number of cases may be a reason for less power and the observed wide CIs. As ascertainment of exposure related to food practices was history based, we cannot completely rule out the chances of misclassification bias. The possibility of recall bias is also very less in this study as most of the information was related to the routine practices of the household members. The estimates for association were adjusted for confounding factors and were conceptualised based on the DAG model. Given the overall low risk of bias, we believe that our findings are valid for the study sample.

Previous estimates suggested that WASH and food practices are associated with enteric fever. A systematic review by Brockett *et al* reported that an improved water source (16 studies; OR 0.73, 95% CI 0.56 to 0.95) and treatment of water before consumption (9 studies; OR 0.59, 95% CI 0.45 to 0.75) are associated with significantly lower odds of typhoid fever. Similar to our findings, it was reported that food and drink consumed outside the home (39 studies) was significantly associated with higher odds of typhoid (OR 1.6, 95% CI 1.4 to 1.8).^5^ The study also reported that that ice cream consumption was significantly associated with higher odds of typhoid (10 studies; OR 1.5, 95% CI 1.2 to 1.9, *I*^2^=31%).^5^ Another study from Kolkata, India, used prospective data from a large cohort to evaluate whether baseline WASH variables predicted typhoid risk in an urban slum area. The analysis reported association with flush toilet (HR=0.30, 95% CI 0.1 to 0.95; p=0.041); source of drinking water from a private tap, well or pump (HR=0.31, 95% CI 0.14 to 0.71; p=0.005); and water treatment by filter or boiling for daily use (HR=0.45, 95% CI 0.18 to 1.1; p=0.081).^23^ Our study findings substantiate reports from previous studies, and therefore it seems that WASH and food hygiene practices are associated with incidence of enteric fever in children in urban low-income neighbourhoods in Delhi.

Our study has certain limitations. As this study was limited to children up to 15 years of age, we cannot comment on the association among adults. We did not include hand hygiene as a risk factor in the present study. It is likely that hand hygiene plays a role in disease transmission among children, and we may have missed this association in the present study. Also, since the study was done in a low socioeconomic neighbourhood of Delhi, it may not be generalisable to other settings.

Our findings suggests that beyond the WASH factors, poor food hygiene practices are associated with enteric fever in children in low-income urban settings in North India. Even though there has been an improvement in WASH parameters over the past few decades, there is a need to focus on improving food hygiene and eating-out practices. Encouragement and involvement of communities to undertake appropriate food hygiene behaviour is a necessary strategy for prevention of enteric infection. The findings also suggest that apart from WASH factors, there may be several other factors that affect the transmission dynamics for, for example, sociodemographic profile and typhoid vaccine coverage. In our population, the vaccination coverage for typhoid vaccine was 13.1%. This highlights the need for strategies to accelerate vaccination coverage beyond improving WASH-related factors. A holistic approach targeted at improving access to safe drinking water and sanitation facilities, education for better food hygiene health practices, disease surveillance and vaccination coverage together may help achieve the goal of disease elimination.

## Data Availability

Data are available upon reasonable request.
